# Appetitive traits and food groups consumption in school-aged children: prospective associations from the Generation XXI birth cohort

**DOI:** 10.1007/s40519-023-01586-9

**Published:** 2023-08-05

**Authors:** Pedro Ferreira, Sarah Warkentin, Andreia Oliveira

**Affiliations:** 1 EPIUnit, Instituto de Saúde Pública, Universidade do Porto [Institute of Public Health, University of Porto], University of Porto, Rua das Taipas nº135, 4050-600 Porto, Portugal; 2 Laboratory for Integrative and Translational Research in Population Health [ITR], University of Porto, Rua das Taipas nº135, 4050-600 Porto, Portugal; 3 Department of Public Health and Forensic Sciences and Medical Education, Faculty of Medicine, University of Porto, Alameda Prof. Hernâni Monteiro, 4200-319 Porto, Portugal

**Keywords:** Feeding behaviour, Appetite, Child, Eating, Cohort studies

## Abstract

**Purpose:**

Appetite can influence children’s dietary choices; however, this relationship in school-aged children is still unclear. We aimed to explore the prospective associations between child appetitive traits at age 7 and food consumption at 10 years of age.

**Methods:**

The study included 3860 children from the Generation XXI birth cohort, recruited in 2005/2006 in Porto, Portugal. The Children’s Eating Behaviour Questionnaire was used to evaluate children’s appetitive traits at 7 years. Food consumption was measured at 10 years through a validated Food Frequency Questionnaire. Logistic regression models were performed and adjusted for possible confounders.

**Results:**

Children with greater Enjoyment of Food at 7 years were 36% more likely to eat fruits ≥ 2 times/day and 54% more likely to eat vegetables > 2.5 times/day at 10 years compared to those with less frequent consumption. Children who ate more in response to negative emotions had higher odds of consuming energy-dense foods (OR = 1.33; 99% CI 1.13–1.58) and salty snacks (OR = 1.28; 99% CI 1.08–1.51) 3 years later. Those with less ability to adjust intake (higher Satiety Responsiveness) and more selective about foods (higher Food Fussiness) at 7 years were less likely to consume vegetables frequently, and were more likely to consume energy-dense foods and sugar-sweetened beverages.

**Conclusions:**

Children’s appetitive traits at 7 years were associated with the consumption of several food groups at 10 years of age. Eating more in response to negative emotions (Emotional Eating), with less ability to adjust intake (Satiety Responsiveness) and more food selectivity (Food Fussiness) were associated with worse dietary choices (in general, lower fruit and vegetables, and higher energy-dense foods and sugar-sweetened beverages consumption).

**Level of evidence:**

Level III: Evidence obtained from well-designed cohort or case–control analytic studies.

**Supplementary Information:**

The online version contains supplementary material available at 10.1007/s40519-023-01586-9.

## Introduction

Appetitive traits are developed early in life and show a long-term stability into adulthood [1, 2]. The modulation of these traits is strongly associated with appetite and implies an interaction between environmental and social factors, as well as internal biological mechanisms [3]. Although the link between appetitive traits and child health indicators, such as body mass index (BMI) [4–6] and fat percentage [7], has been established in previous studies, the relationship between appetite and later dietary intake in school-aged children has rarely been explored in prospective studies.

Appetitive traits during childhood may vary from disinhibited eating to picky eating with different short- and long-term implications on health, such as increased obesity risk and/or poor dietary variety [8, 9]. It has been described that certain features of appetitive traits are associated with overeating and overweight [10, 11]. In this context, children showing high responsiveness to external food cues and low sensitivity to internal satiety (fullness) cues, develop an avid appetite and may overconsume calories in relation to energy needs [10]. In contrast, appetitive traits in which the child eats a limited amount of food and/or is unwilling to try new foods are also relatively common [12]. Picky eaters typically show persistent food refusal resulting in a lower dietary diversity [13] and a lower intake of specific foods, such as vegetables [14, 15]. These strong food preferences often lead parents to provide the child with different food options from those eaten by other family members [14].

Evidence on how certain appetitive traits affect food preferences and, probably, dietary consumption in school-aged children is supported by a limited number of prospective studies. A study in 9-year-old Dutch children found that food approach traits, which are related to a greater interest towards foods, such as Enjoyment of Food and Food Responsiveness, were positively associated with fruit consumption, after a 1-year follow-up, while food avoidant traits, which are linked to a lack of interest towards foods and high selectivity, such as Food Fussiness, were negatively associated with later fruit intake [16]. Nevertheless, food avoidant traits, which are thought to be ‘protective’ for obesity, such as Satiety Responsiveness and Slowness in Eating, were associated with greater snacks and sugar-sweetened beverages intake after 1 year, respectively, and Enjoyment of Food was associated with lower snacking frequency [16]. In another prospective study with younger children, the same trend was described between appetitive traits measured at 16 months of age and later fruit and vegetables preference (at 3–4 years). Food approach traits were positively and food avoidant traits were negatively associated with later fruit and vegetables preferences [17]. The authors also concluded that Food Responsiveness was related to a higher preference for noncore foods (i.e., foods high in fat and/or sugar and energy density) [17]. According to cross-sectional studies, children showing greater scores on Enjoyment of Food and Food Responsiveness at 6–8 years are more likely to consume a wider variety of foods, both protein-rich foods, such as meat, and nutrient-rich foods, such as vegetables, fruit and berries [18]. On the other hand, children scoring higher in food avoidant traits, such as Food Fussiness and Satiety Responsiveness, were less likely to consume vegetables and fruit [18, 19], but were more likely to show a greater consumption of snack foods [19].

To improve children’s diet quality along with adequate growth, it is a necessary step to assess appetitive traits and understand their contribution to food consumption over time. These associations have been narrowly studied in school-aged children, particularly through prospective studies. To the best of our knowledge, the majority of prospective studies focused on restricted food groups, such as fruit and vegetables, which gathers the strongest evidence, but other food groups, such as energy-dense foods, dairy products, starchy foods, meat and fishery, were less explored and findings are inconsistent. To address this research gap, the present study aims to investigate the prospective association between child appetitive traits at 7 years and food groups consumption at 10 years of age. We hypothesize that those children with greater scores on food approach traits would show a greater intake of several food groups, including energy-dense foods, 3 years later. We also expect to find a negative association between child food avoidant traits at 7 years and the consumption of fruit and vegetables and a positive association with noncore foods 3 years later.

## Methods

### Participants and study design

The current study included children from the Generation XXI, an ongoing population-based birth cohort previously described [20, 21]. Participants were recruited at delivery, between April 2005 and August 2006, in all public maternity units in the Metropolitan Area of Porto [Northern Portugal]. At birth, 91.4% of the invited mothers agreed to participate (*n* = 8495 mothers and 8647 children). Follow-ups occurred when children were 4, 7 and 10 years of age, achieving a participation proportion of 86%, 80%, and 74%, respectively.

**Fig. 1:**
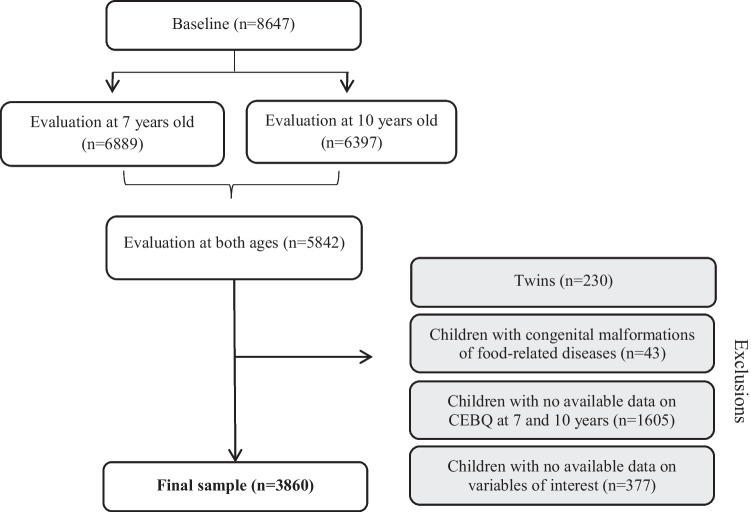
Study flowchart of participants from the Generation XXI birth cohort

The present study includes data from the follow-up at 7 and 10 years of age. Twins (*n* = 230) and children with congenital anomalies or diseases that might influence dietary intake (e.g., celiac disease, food allergy, food intolerance and phenylketonuria) (*n* = 43) were excluded from the current analysis. In addition, participants with no information on appetitive traits at 7 and 10 years (*n* = 1605) and those with missing data on variables of interest (*n* = 377) were excluded. After exclusions, the current sample consisted of 3860 children (see study flowchart in Fig. 1).

### Ethics

Generation XXI was approved by the University of Porto Medical School/São João Hospital Centre Ethics Committee (27 April 2005) and by the Portuguese Data Protection Authority (Protocol code 5833, approved on 30 May 2011). All the phases of the study complied with the Ethical Principles for Medical Research Involving Human Subjects expressed in the Declaration of Helsinki. A written informed consent from the parents (or legal substitute) and an oral assent from the children were obtained in each follow-up.

### Mother and child characteristics

Information on socio-demographic characteristics, mother's prenatal care, health and lifestyles were collected at baseline by trained researchers, through face-to-face interviews using structured questionnaires, or retrieved from medical records. Data on maternal age, education, smoking habits during pregnancy, child’s sex, birth weight and gestational age were included in the current study. Maternal BMI was calculated from self-reported pre-pregnancy weight and height and categorized accordingly [22]. Child’s anthropometric assessment was performed at 7 and 10 years following standard procedures [23], and BMI z-scores were calculated based on age and sex-specific standards, following the World Health Organization (WHO) Growth Charts [24]. Child’s weight status was categorized into “underweight” [< − 2 standard deviation (SD)], “normal weight” (− 2 ≤ SD ≤ 1), “overweight” (1 < SD ≤ 2), and “obesity” (> 2 SD) for descriptive purposes only. Regular and scheduled practice of sports at school or out of school and living with siblings was evaluated at the age of 7 through a dichotomous question (yes/no).

### Food consumption

Food consumption was evaluated at 10 years of age through a 41-item qualitative Food Frequency Questionnaire (FFQ), having as reference the period of 6 months previous to the research interview. The child's primary  caregiver had nine response options to each food item, varying from “≥ 4 times per day” to “< once a month or never”. Frequencies of food consumption were converted into daily frequencies (e.g., once a week was converted into 1/7 days = 0.14 times/day) or weekly frequencies (e.g., 5–6 times/week was converted into the mean of 5.5 times/week) for statistical purposes. Fourteen food groups were defined based on similar nutritional composition and food type, namely, vegetables (cooked or raw vegetables and vegetable soup), fruit (fresh), milk (milk and chocolate milk), yoghurt, cheese, meat (all types of meat), fishery (all types of fish and shellfish), eggs, meat products (ham, sausages and smoked meat products), rice, potatoes and pasta, breakfast cereals, sugar-sweetened beverages (carbonated and non-carbonated sugar-sweetened beverages), energy-dense foods (pizza, chips, chocolate, cakes, pastry, cookies, candies) and salty snacks (meat and fish-based fried snacks). Consumption frequencies were defined as times/day or times/week, as appropriate, and grouped into two or three categories using, as cut-offs, sample medians or tertiles, respectively. The current FFQ showed acceptable validity to estimate food consumption, when compared with 3-day food records among a subsample of children from the Generation XXI cohort at 4 and 7 years of age [25], and was previously adapted for use at age 10 [26].

### Appetitive traits

Appetitive traits were assessed at the 7-year-old follow-up using the Portuguese version of the Children’s Eating Behaviour Questionnaire (CEBQ) [27], originally developed in the UK by Wardle et al. [28]. The questionnaire is composed by 35 items, which were answered by the main caregiver (94% were mothers) using a five-point Likert scale, ranging from 1—“never” to 5—“always”. These items are organized into eight appetitive subscales; four measuring child`s general appetite and interest towards foods and drinks, and were, therefore, referred to as food approach traits: Food Responsiveness (FR, 5 items), Enjoyment of Food (EF, 4 items), Emotional Overeating (EOE, 4 items) and Desire to Drink (DD, 3 items). The other four measure child’s lack of interest and avoidance towards foods, and were, therefore, referred to as food avoidant traits: Satiety Responsiveness (SR, 5 items), Slowness in Eating (SE, 4 items), Emotional Undereating (EUE, 4 items) and Food Fussiness (FF, 6 items). Items in each subscale were summed and its mean scores were calculated. Greater scores of each subscale indicate higher levels of each appetitive trait. As in the original validation study [28], five items were reverse-coded due to opposite phrasing. In questionnaires with < 50% of missing items (approximately 3%), data were imputed by replacing the average of the remaining questions within each subscale of the participant. Adequate psychometric properties of the CEBQ were observed at 7 years, with Cronbach`s alphas [α] ranging from 0.74 to 0.85 [27] and in the current sample (α = 0.77–0.85). In addition, we calculated McDonald’s Omega [29, 30], with values ranging from 0.78 to 0.85, confirming a good internal consistency of all CEBQ subscales.

### Statistical analysis

Participant’s characteristics are described as counts (n) and percentages (%) for categorical variables, and as mean (M) and standard deviation (SD), or median (Md) and interquartile ranges (IQR), depending on the distribution of continuous variables.

The associations between appetitive traits subscales at 7 years and food groups consumption at 10 years were evaluated through binary (when food groups were dichotomized by their median) or multinomial logistic regression models (when food groups were categorized according to tertiles), considering the lowest consumption as the reference category. Associations are described for each unit increase in each appetitive traits subscale score. Different models were compared considering potential confounding factors, such as maternal age, education and pre-pregnancy BMI, which were selected based on literature review [31–33], plus child’s sex and sports practice and child’s BMI z-score due to the possible associations with dietary intake [34] and appetitive traits [35]. Aiming to control for the potential cross-sectional effects on food consumption at 10 years of age, models were further adjusted for the respective food group consumption at 7 years, and also for each CEBQ subscale at 10 years of age. Based on this, the following models were estimated: model 0 (crude model); model 1 (adjusted for mother’s age, education and pre-pregnancy BMI, child’s sex, sports practice at 7 years of age and child BMI z-score at 10). Model 2 was further adjusted for food consumption at 7 years and CEBQ subscale at 10 years, and is described in Supplementary table 1a, b. Interaction effects of child's sex and BMI z-score in these associations were tested by adding an interaction term into the adjusted model 1. Nevertheless, no significant interaction effects were found, so interaction terms were not included in the final models.

Results are presented as *Odds Ratios* (OR) and the respective 99% confidence intervals (99%CI). An alpha of 1% was considered for statistical significance to correct for multiple comparisons. Statistical analyses were performed using SPSS Statistics version 25 software (IBM Corp. Released 2017. IBM SPSS Statistics for Windows, Version 25.0. Armonk, NY: IBM Corp.).

## Results

Mother and child characteristics are described in Table 1. At baseline, mothers had a mean age of 30 years (SD = 5.1), and a median of 12 years of schooling (IQR = 7.0). At 7 years of age, 38.9% of children were classified as having overweight or obesity, 60.7% were living with siblings and the majority reported having a regular and scheduled practice of sports, including at school and outside school (86.2%). Frequency of food groups consumption at 10 years are described in Table 2.

**Table 1:** Mother and child characteristics at baseline and 7-year follow-up (*n* = 3860)

Mother characteristics	*n* (%), M (SD) or Md (IQR)
Age (years) at baseline, M (SD)	30.0 (5.1)
Education (years) at baseline, Md (IQR)	12.0 (7.0)
Pre-pregnancy BMI (kg/m^2^), Md (IQR)	23.0 (4.7)
Smoking during pregnancy, n (%)	
No	3083 (80.8)
Yes	731 (19.2)
**Child characteristics**	
Sex, n (%)	
Girls	1872 (48.5)
Boys	1988 (51.5)
Gestational age (weeks), Md (IQR)	39.0 (2.0)
Birth weight (kg), M (SD)	3.20 (0.48)
BMI z-score at 7 years, Md (IQR)	0.6 (1.60)
Weight status at 7 years, n (%)^a^	
Underweight (< -2 SD)	10 (0.3)
Normal weight (− 2 ≤ SD ≤ 1)	2351 (60.9)
Overweight (1 < SD ≤ 2)	960 (24.9)
Obesity (> 2 SD)	539 (14.0)
Living with siblings at 7 years, n (%)	
No	1503 (39.3)
Yes	2324 (60.7)
Sports practice at 7 years, n (%)	
No	534 (13.8)
Yes	3326 (86.2)
Child appetitive traits (CEBQ, possible range 1–5) at 7 years	M (SD)
Enjoyment of Food	3.01 (0.79)
Food Responsiveness	2.04 (0.78)
Emotional Overeating	1.82 (0.63)
Desire to Drink	2.21 (0.81)
Satiety Responsiveness	2.71 (0.69)
Slowness in Eating	2.93 (0.88)
Emotional Undereating	2.46 (0.76)
Food Fussiness	2.96 (0.76)

*M* Mean, *Md* Median, *SD* Standard deviation, *IQR* Interquartile range, *BMI* Body mass index, *CEBQ* Children's Eating Behaviour Questionnaire

aWeight status defined using BMI z-scores, according to the WHO Growth Charts [24]

**Table 2:** Description of parent-reported food consumption frequencies at 10 years of age (*n* = 3860)

Frequency of food groups consumption at 10 years*		*n* (%)
Vegetables^a^	< 1.5 times/day	1199 (31.1)
	1.5–2.5 times/day	1367 (35.4)
	> 2.5 times/day	1294 (33.5)
Fruit	< 2 times/day	1974 (51.1)
	≥ 2 times/day	1886 (48.9)
Milk^b^	< 1 times/day	686 (17.8)
	1–2 times/day	1757 (45.5)
	> 2 times/day	1417 (36.7)
Yoghurt	< 0.5 times/day	1783 (46.2)
	0.5–1 times/day	2077 (53.8)
Cheese	< 1 times/week	1135 (29.4)
	1–3 times/week	1723 (44.6)
	> 3 times/week	1002 (26.0)
Meat^c^	< 2 times/week	1210 (31.3)
	2–4 times/week	1557 (40.3)
	> 4 times/week	1093 (28.3)
Fishery^d^	< 3 times/week	1967 (51.0)
	≥ 3 times/week	1893 (49.0)
Eggs	≤ 1 time/day	2415 (62.6)
	> 1 time/day	1445 (37.4)
Meat Products^e^	< 2 times/week	1210 (31.3)
	2–4 times/week	1557 (40.3)
	> 4 times/week	1093 (28.3)
Rice, potatoes and pasta	< 2 times/day	353 (9.1)
	≥ 2 times/day	3507 (90.9)
Breakfast cereals	< 2 times/week	1171 (30.3)
	2–5 times/week	1162 (30.1)
	≥ 6 times/week	1527 (39.6)
Sugar-sweetened beverages^f^	< 0.5 times/day	1294 (33.5)
	0.5–1 times/day	1036 (26.8)
	> 1 time/day	1530 (39.6)
Energy-dense foods^g^	< 1.3 times/day	1431 (37.1)
	1.3–2 times/day	1255 (32.5)
	> 2 times/day	1174 (30.4)
Salty snacks^h^	< 0.6 times/day	1340 (34.7)
	0.6–1 times/day	1261 (32.7)
	> 1 time/day	1259 (32.6)

*Sample median or tertiles were used to define cut-off points; ^a^Includes cooked or raw vegetables, and vegetable soup; ^b^Includes milk and chocolate milk; ^c^Includes all types of meats, excluding meat products; ^d^Includes all types of fish and shellfish ^e^Includes ham, sausages and smoked meat products; ^f^Includes carbonated and non-carbonated sugar-sweetened beverages; ^g^Includes pizza, burgers, chips, chocolate, snacks, cakes, pastry, cookies and candies; ^h^Includes meat and fish-based fried snacks

Binary and multinomial logistic regressions with associations between appetitive traits at 7 years and food groups consumption at age 10 are presented in Table 3 (Crude models) and Table 4 (Models 1). According to the multivariable analyses, children showing greater scores on Enjoyment of Food at 7 years had significantly higher odds of consuming vegetables (> 2.5 times/day: OR = 1.54, 99% CI 1.33–1.79), fruit (≥ 2 times/day: OR = 1.36, 99% CI 1.20–1.53) and fishery (≥ 3 times/week: OR = 1.15, 99% CI 1.02–1.29) more frequently, compared to those with lower frequency of intake of each food group, 3 years later (Table 4). In addition, children with higher scores on Enjoyment of Food at 7 also showed lower odds of drinking milk more than 2 times/day (OR = 0.78, 99% CI 0.66–0.93) and sugar-sweetened beverages more than once a day (OR = 0.83, 99% CI 0.73–0.96) 3 years later, compared to those with lower intake frequencies of each of these food groups (Table 4). Children with higher scores on Food Responsiveness at 7 years were 19% more likely to consume fruit ≥ 2 times/day and 20% more likely to consume energy-dense foods > 2 times/day at 10 years, when compared to those with lower intake frequencies. Higher scores on Food Responsiveness at 7 years were also inversely associated with sugar-sweetened beverages intake (0.5–1 times/day: OR = 0.85, 99% CI 0.73–0.99), but positively associated with the consumption of energy-dense foods (> 2 times/day: OR = 1.20, 99% CI 1.04–1.38) and fruit (≥ 2 times/day: OR = 1.19, 99% CI 1.06–1.34) at age 10. Children eating more in response to negative emotions at 7 years (i.e., greater scores on Emotional Overeating) showed greater odds of consuming more frequently energy-dense foods and salty snacks at 10 years, compared with those in the lower food consumption group. Higher scores on Emotional Overeating were also positively associated with meat, meat products and breakfast cereals consumption at age 10. Children who showed higher scores on Desire to Drink at 7 years, were less likely to consume vegetables (> 2.5 times/day: OR = 0.80; 99% CI 0.70–0.91) and fishery (≥ 3 times/week: OR = 0.89; 99% CI 0.80–0.99), when compared with those with a less frequent intake, 3 years later. On the other hand, these children were more likely to consume non-core foods and drink sugar-sweetened beverages more frequently at age of 10 (Table 4).

**Table 3:** Binary and multinomial logistic regressions for associations between child appetitive traits at 7 years (for each unit increase in each subscale score) and food consumption at 10 years of age—Crude Models (*n* = 3860)

Food consumption at 10 years		Appetitive traits at 7 years							
		Enjoyment of Food	Food Responsiveness	Emotional Overeating	Desire to Drink	Satiety Responsiveness	Slowness in Eating	Emotional Undereating	Food Fussiness
		OR (99% CI)	OR (99% CI)	OR (99% CI)	OR (99% CI)	OR (99% CI)	OR (99% CI)	OR (99% CI)	OR (99% CI)
Vegetables^a^	1.5–2.5 times/day	1.10 (0.97–1.25)	0.98 (0.86–1.11)	1.03 (0.87–1.21)	**0.86 (0.76–0.97)**	0.91 (0.79–1.05)	1.01 (0.90–1.13)	1.03 (0.90–1.18)	**0.64 (0.56–0.74)**
Ref: < 1.5 times/day	> 2.5 times/day	**1.23 (1.08–1.40)**	0.96 (0.84–1.10)	0.88 (0.74–1.04)	**0.72 (0.63–0.82)**	**0.71 (0.61–0.83)**	0.98 (0.87–1.10)	0.91 (0.80–1.05)	**0.42 (0.37–0.49)**
Fruit	≥ 2 times/day	**1.21 (1.09–1.35)**	1.11 (1.00–1.24)	1.02 (0.89–1.16)	0.92 (0.83–1.02)	**0.83 (0.74–0.94)**	0.93 (0.85–1.03)	0.99 (0.89–1.11)	**0.73 (0.65–1.81)**
Ref: < 2 times/day									
Milk^b^	1–2 times/day	**0.86 (0.74–0.99)**	0.90 (0.78–1.04)	0.89 (0.74–1.07)	1.04 (0.90–1.20)	1.11 (0.94–1.32)	**1.19 (1.04–1.37)**	0.98 (0.84–1.14)	1.09 (0.94–1.27)
Ref: < 1 time/day	> 2 times/day	**0.77 (0.66–0.90)**	**0.83 (0.71–0.97)**	0.92 (0.76–1.11)	1.08 (0.93–1.26)	**1.37 (1.14–1.63)**	**1.40 (1.22–1.61)**	1.05 (0.89–1.23)	**1.18 (1.01–1.39)**
Yoghurt	0.5–1 times/day	1.05 (0.95–1.17)	1.07 (0.96–1.19)	1.10 (0.96–1.26)	0.95 (0.86–1.05)	**0.87 (0.77–0.98)**	0.94 (0.85–1.03)	0.94 (0.84–1.05)	0.97 (0.87–1.08)
Ref: < 1 time/day									
Cheese	1–3 times/week	**1.16 (1.03–1.32)**	1.14 (1.00–1.30)	1.09 (0.93–1.28)	0.97 (0.86–1.10)	**0.86 (0.75–0.99)**	0.97 (0.87–1-09)	1.02 (0.90–1.16)	**0.70 (0.61–0.79)**
Ref: < 1 time/week	> 3 times/week	1.14 (0.99–1.31)	1.10 (0.95–1.27)	1.09 (0.91–1.30)	0.97 (0.84–1.11)	0.89 (0.76–1.05)	1.06 (0.93–1.20)	1.01 (0.87–1.17)	**0.74 (0.63–0.85)**
Meat ^c^	2–4 times/week	1.10 (0.97–1.25)	1.09 (0.96–1.24)	1.13 (0.96–1.33)	1.11 (0.98–1.26)	0.93 (0.81–1.08)	0.95 (0.85–1.07)	0.99 (0.87–1.13)	0.96 (0.84–1.09)
Ref: < 2 times/week	> 4 times/week	**1.17 (1.02–1.34)**	**1.18 (1.03–1.35)**	**1.28 (1.08–1.52)**	**1.23 (1.08–1.41)**	0.94 (0.81–1.10)	0.99 (0.87–1.11)	1.02 (0.88–1.17)	0.93 (0.80–1.06)
Fishery^d^	≥ 3 times/week	1.07 (0.96–1.18)	0.94 (0.84–1.05)	0.89 (0.78–1.01)	**0.84 (0.76–0.93)**	0.89 (0.79–1.00)	0.99 (0.90–1.09)	**0.87 (0.78–0.97)**	**0.76 (0.68–0.85)**
Ref: < 3 times/week									
Eggs	> 1 time/day	1.00 (0.90–1.12)	0.97 (0.86–1.08)	1.04 (0.91–1.19)	0.91 (0.82–1.02)	1.00 (0.88–1.13)	0.96 (0.87–1.05)	1.11 (0.99–1.24)	**0.87 (0.78–0.98)**
Ref: ≤ 1 time/day									
Meat Products^e^	2–4 times/week	1.10 (0.97–1.25)	1.09 (0.96–1.24)	1.13 (0.96–1.33)	1.11 (0.98–1.26)	0.93 (0.81–1.08)	0.95 (0.85–1.07)	0.99 (0.87–1.13)	0.96 (0.84–1.09)
Ref: ≤ 2 times/week	> 4 times/week	**1.17 (1.02–1.34)**	**1.18 (1.03–1.35)**	**1.28 (1.08–1.52)**	**1.23 (1.08–1.41)**	0.94 (0.81–1.10)	0.99 (0.87–1.11)	1.02 (0.88–1.17)	0.93 (0.80–1.06)
Rice, potatoes and pasta	≥ 2 times/day	0.88 (0.74–1.06)	0.90 (0.75–1.07)	0.91 (0.72–1.13)	1.10 (0.92–1.32)	1.09 (0.88–1.34)	0.97 (0.82–1.14)	0.97 (0.80–1.17)	1.04 (0.86–1.26)
Ref: < 2 times/day									
Breakfast Cereals	2–5 times/week	1.01 (0.88–1.15)	1.01 (0.89–1.16)	1.15 (0.96–1.36)	1.02 (0.89–1.16)	1.02 (0.87–1.19)	1.11 (0.98–1.25)	1.01 (0.88–1.16)	0.91 (0.80–1.05)
Ref: < 2 times/week	> 6 times/week	0.96 (0.85–1.09)	0.98 (0.86–1.12)	1.14 (0.97–1.35)	1.07 (0.95–1.22)	1.08 (0.93–1.24)	1.10 (0.98–1.23)	1.08 (0.95–1.23)	1.01 (0.89–1.15)
Sugar-sweetened beverages^f^	0.5–1 times/day	0.93 (0.81–1.07)	0.93 (0.81–1.06)	0.98 (0.82–1.16)	1.07 (0.93–1.23)	1.11 (0.95–1.30)	0.99 (0.88–1.12)	1.12 (0.97–1.29)	0.99 (0.86–1.14)
Ref: < 0.5 times/day	> 1 time/day	0.92 (0.81–1.04)	0.94 (0.83–1.07)	1.15 (0.98–1.34)	**1.40 (1.24–1.59)**	**1.18 (1.03–1.36)**	1.05 (0.94–1.17)	1.09 (0.96–1.24)	1.11 (0.98–1.27)
Energy-dense foods^g^	1.3–2 times/day	0.91 (0.80–1.03)	1.01 (0.89–1.15)	1.18 (1.00–1.38)	1.11 (0.97–1.26)	1.09 (0.94–1.26)	1.07 (0.95–1.19)	1.15 (1.00–1.31)	**1.19 (1.04–1.35)**
Ref: < 1.3 times/day	> 2 times/day	**0.85 (0.75–1.97)**	1.06 (0.93–1.21)	**1.25 (1.06–1.47)**	**1.32 (1.16–1.50)**	**1.43 (1.23–1.66)**	**1.16 (1.03–1.30)**	**1.21 (1.06–1.38)**	**1.30 (1.14–1.49)**
Salty snacks^h^	0.6–1 times/day	1.02 (0.90–1.16)	1.07 (0.94–1.21)	1.14 (0.97–1.35)	1.07 (0.94–1.22)	0.92 (0.80–1.07)	0.89 (0.79–1.00)	1.06 (0.93–1.21)	0.94 (0.82–1.07)
Ref: < 0.6 times/week	> 1 time/day	0.97 (0.85–1.10)	1.04 (0.91–1.18)	**1.28 (1.09–1.50)**	**1.23 (1.08–1.39)**	1.03 (0.89–1.19)	0.96 (0.86–1.08)	**1.15 (1.01–1.32)**	**1.18 (1.04–1.35)**

^a^Includes cooked or raw vegetables, and vegetable soup; ^b^Includes milk and chocolate milk; ^c^Includes all types of meats, excluding meat products; ^d^Includes all types of fish and shellfish;  ^e^Includes ham, sausages and smoked meat products; ^f^Includes carbonated and non-carbonated sugar-sweetened beverages; ^g^Includes pizza, burgers, chips, chocolate, snacks, cakes, pastry, cookies and candies; ^h^Includes meat and fish-based fried snacks. Ref.: Reference category; OR: Odds Ratio; 99% CI 99% Confidence Interval. Significant associations (*p* < 0.01) are highlighted in bold type

**Table 4:** Binary and multinomial logistic regressions for associations between child appetitive traits at 7 years (for each unit increase in each subscale score) and food consumption at 10 years of age—Models 1 (*n* = 3860)

Food consumption at 10 years		Appetitive traits at 7 years							
		Enjoyment of Food	Food Responsiveness	Emotional Overeating	Desire to Drink	Satiety Responsiveness	Slowness in Eating	Emotional Undereating	Food Fussiness
		OR (99% CI)	OR (99% CI)	OR (99% CI)	OR (99% CI)	OR (99% CI)	OR (99% CI)	OR (99% CI)	OR (99% CI)
Vegetables^a^	1.5–2.5 times/day	**1.20 (1.04–1.39)**	1.02 (0.88–1.16)	1.06 (0.90–1.25)	0.90 (0.79–1.02)	**0.84 (0.71–0.98)**	0.97 (0.86–1.11)	0.99 (0.87–1.14)	**0.59 (0.51–0.69)**
Ref: < 1.5 times/day	> 2.5 times/day	**1.54 (1.33–1.79)**	1.08 (0.93–1.26)	0.96 (0.80–1.14)	**0.80 (0.70–0.91)**	**0.57 (0.48–0.67)**	0.90 (0.79–1.03)	**0.83 (0.72–0.96)**	**0.37 (0.31–0.43)**
Fruit	≥ 2 times/day	**1.36 (1.20–1.53)**	**1.19 (1.06–1.34)**	1.07 (0.93–1.23)	1.00 (0.90–1.12)	**0.76 (0.67–87)**	0.92 (0.82–1.02)	0.94 (0.84–1.06)	**0.70 (0.62–0.78)**
Ref: < 2 times/day									
Milk^b^	1–2 times/day	**0.83 (0.71–0.98)**	0.90 (0.77–1.06)	0.90 (0.74–1.08)	1.01 (0.87–1.17)	1.13 (0.94–1.36)	**1.24 (1.07–1.44)**	0.99 (0.84–1.15)	1.09 (0.93–1.27)
Ref: < 1 time/day	> 2 times/day	**0.78 (0.66–0.93)**	0.86 (0.73–1.02)	0.95 (0.78–1.15)	1.04 (0.89–1.22)	**1.35 (1.12–1.63)**	**1.43 (1.23–1.67)**	1.05 (0.89–1.23)	1.17 (1.00–1.37)
Yoghurt	0.5–1 times/day	1.00 (0.89–1.12)	1.03 (0.91–1.15)	1.07 (0.93–1.23)	0.91 (0.82–1.02)	0.90 (0.79–1.03)	0.97 (0.88–1.08)	0.96 (0.86–1.07)	0.99 (0.89–1.11)
Ref: < 1 time/day									
Cheese	1–3 times/week	1.11 (0.97–1.27)	1.08 (0.94–1.25)	1.05 (0.89–1.24)	0.97 (0.86–1.10)	0.91 (0.78–1.06)	1.03 (0.91–1.16)	1.04 (0.91–1.18)	**0.71 (0.62–0.81)**
Ref: < 1 time/week	> 3 times/week	1.10 (0.94–1.28)	1.05 (0.89–1.23)	1.05 (0.87–1.26)	0.98 (0.85–112)	0.93 (0.78–1.10)	1.12 (0.98–1.29)	1.02 (0.88–1.18)	**0.72 (0.64–0.86)**
Meat^c^	2–4 times/week	1.05 (0.91–1.21)	1.04 (0.90–1.19)	1.09 (0.92–1.28)	1.05 (0.92–1.19)	0.98 (0.84–1.15)	0.98 (0.87–1.11)	1.03 (0.90–1.17)	0.98 (0.86–1.12)
Ref: < 2 times/week	> 4 times/week	1.10 (0.95–1.28)	1.11 (0.95–1.29)	**1.21 (1.01–1.44)**	1.15 (1.00–1.31)	1.01 (0.86–1.20)	1.05 (0.92–1.19)	1.06 (0.92–1.23)	0.95 (0.82–1.10)
Fishery^d^	≥ 3 times/week	**1.15 (1.02–1.29)**	0.96 (0.86–1.09)	0.91 (0.79–1.04)	**0.89 (0.80–0.99)**	**0.82 (0.72–0.94)**	0.96 (0.87–1.07)	**0.83 (0.74–0.93)**	**0.74 (0.66–0.83)**
Ref: < 3 times/week									
Eggs	> 1 time/day	1.07 (0.95–1.21)	1.02 (0.91–1.16)	1.09 (0.95–1.26)	0.96 (0.86–1.07)	0.96 (0.84–1.09)	0.92 (0.83–1.03)	1.08 (0.97–1.22)	**0.85 (0.76–0.96)**
Ref: ≤ 1 time/day									
Meat Products^e^	2–4 times/week	1.05 (0.91–1.21)	1.04 (0.90–1.19)	1.09 (0.92–1.28)	1.05 (0.92–1.19)	0.98 (0.84–1.15)	0.98 (0.87–1.11)	1.03 (0.90–1.17)	0.98 (0.86–1.12)
Ref: ≤ 2 times/week	> 4 times/week	1.10 (0.95–1.28)	1.11 (0.95–1.29)	**1.21 (1.01–1.44)**	1.15 (1.00–1.31)	1.01 (0.86–1.20)	1.05 (0.92–1.19)	1.06 (0.92–1.23)	0.95 (0.82–1.10)
Rice, potatoes and pasta	≥ 2 times/day	0.91 (0.74–1.11)	0.92 (0.76–1.12)	0.92 (0.73–1.15)	1.07 (0.88–1.29)	1.04 (0.83–1.31)	0.92 (0.77–1.10)	0.97 (0.80–1.18)	1.03 (0.85–1.24)
Ref: < 2 times/day									
Breakfast Cereals	2–5 times/week	1.07 (0.92–1.24)	1.08 (0.93–1.26)	1.19 (1.00–1.42)	1.01 (0.88–1.15)	0.97 (0.82–1.15)	1.07 (0.94–1.23)	1.01 (0.87–1.16)	0.90 (0.78–1.03)
Ref: < 2 times/week	> 6 times/week	1.08 (0.94–1.24)	1.11 (0.97–1.28)	**1.23 (1.04–1.46)**	1.04 (0.92–1.18)	0.97 (0.83–1.13)	1.01 (0.90–1.15)	1.07 (0.94–1.23)	0.98 (0.85–1.12)
Sugar-sweetened beverages^f^	0.5–1 times/day	**0.85 (0.73–0.99)**	**0.85 (0.73–0.99)**	0.94 (0.78–1.12)	1.03 (0.89–1.18)	**1.22 (1.03–1.44)**	1.04 (0.91–1.19)	1.16 (1.00–1.34)	1.01 (0.88–1.17)
Ref: < 0.5 times/day	> 1 time/day	**0.83 (0.73–0.96)**	0.87 (0.76–1.01)	1.10 (0.94–1.30)	**1.27 (1.12–1.45)**	**1.29 (1.10–1.51)**	1.07 (0.95–1.21)	**1.16 (1.01–1.33)**	1.15 (1.00–1.31)
Energy-dense foods^g^	1.3–2 times/day	0.93 (0.81–1.07)	1.05 (0.91–1.21)	**1.20 (1.02–1.42)**	1.08 (0.95–1.23)	1.06 (0.91–1.24)	1.03 (0.91–1.16)	**1.15 (1.01–1.32)**	**1.18 (1.03–1.34)**
Ref: < 1.3 times/day	> 2 times/day	0.92 (0.80–1.06)	**1.20 (1.04–1.38)**	**1.33 (1.13–1.58)**	**1.29 (1.13–1.47)**	**1.34 (1.15–1.58)**	1.07 (0.94–1.21)	**1.21 (1.06–1.39)**	**1.27 (1.11–1.46)**
Salty snacks^h^	0.6–1 times/day	0.99 (0.86–1.14)	1.03 (0.89–1.18)	1.12 (0.95–1.32)	1.04 (0.91–1.19)	0.93 (0.79–1.09)	0.88 (0.78–1.00)	1.07 (0.93–1.22)	0.95 (0.83–1.08)
Ref: < 0.6 times/week	> 1 time/day	0.95 (0.83–1.09)	1.04 (0.90–1.20)	**1.28 (1.08–1.51)**	**1.19 (1.04–1.35)**	1.03 (0.88–1.21)	0.94 (0.83–1.06)	**1.18 (1.03–1.35)**	1.19 (1.04–1.37)

Model 1—Adjusted for mother’s age, education, pre-pregnancy body mass index, child’s sex, sports practice and BMI z-score at 10 years

^a^Includes cooked or raw vegetables, and vegetable soup; ^b^Includes milk and chocolate milk; ^c^Includes all types of meats, excluding meat products; ^d^Includes all types of fish and shellfish; ^e^Includes ham, sausages and smoked meat products; ^f^Includes carbonated and non-carbonated sugar-sweetened beverages; ^g^Includes pizza, burgers, chips, chocolate, snacks, cakes, pastry, cookies and candies; ^h^Includes meat and fish-based fried snacks. Ref.: Reference category; OR: Odds Ratio; 99% CI 99% Confidence Interval. Significant associations (*p* < 0.01) are highlighted in bold type

Regarding food avoidant traits, children with greater scores on Satiety Responsiveness and Food Fussiness at 7 years had significantly lower odds of consuming vegetables > 2.5 times/day at 10 years, compared to those consuming < 1.5 times/day (OR = 0.57; 99% CI 0.48–0.67 and OR = 0.37; 99% CI 0.31–0.43, respectively) at 10 years. These appetitive traits were also associated with lower consumption frequency of fruit and fishery at 10 years. In contrast, children with higher scores on Satiety Responsiveness at 7 years (i.e., showing higher ability to recognize and adjust food intake in response to internal signals) showed to be 34% more likely to consume energy-dense foods > 2 times/day, 29% more likely to drink sugar-sweetened beverages > 1 time/day, and 35% more likely to consume milk > 2 times/day at 10 years, compared to those with the lowest consumption frequencies of these food groups (Table 4). These children also showed lower odds of consuming vegetables, fruit, and fishery more frequently at 10 years. Children who tend to eat less in response to negative emotions (i.e., greater scores on Emotional Undereating) at 7 years were more likely to consume more frequently energy-dense foods, salty snacks and sugar-sweetened beverages, and less likely to consume vegetables and fishery at 10 years, compared with those in the lower food consumption groups. Finally, children who eat slowly at 7 years had significantly higher odds of consuming milk > 2 times/day at 10 years, compared to those consuming < 1 time/day (OR = 1.43; 99% CI 1.23–1.67).

After further adjusting the models for the respective food consumption group at 7 years of age and the respective CEBQ subscale at 10 years, significant results were weakened or lost statistical significance (see Supplementary Table 1a, b). Overall, it seems that part of the observed effects might be explained by the food consumption at 7 years and/or by the appetitive behaviour at 10 years.

## Discussion

This study examined the prospective associations between children’s appetitive traits and later food consumption and, to our knowledge, it is one of the few studies that analysed this relationship in a variety of foods, instead of focusing on a specific food group. The results suggest that appetitive traits of 7 years are associated with food consumption at age 10. For some food groups, the associations seem to be influenced by the child’s food consumption at 7 years and/or appetitive traits at 10 years.

As predicted, children with greater interest towards foods and who enjoy eating showed higher consumption frequency of several food groups 3 years later, including vegetables, fruit, and also energy-dense foods. Similarly, in a previous study with children  from the UK and Australia, Fildes et al. [17] concluded that children with greater scores on food approach traits in early childhood (at 16 months of age) and preschool-age (3-4 years), namely, Enjoyment of Food and Food Responsiveness, showed greater liking for vegetables and fruit (Enjoyment of Food trait) and noncore foods (Food Responsiveness trait). Other studies that assessed the associations between appetitive traits and food consumption using cross-sectional designs have replicated our results [18, 36]. In Finish children aged 6–8 years, Enjoyment of Food and Food Responsiveness were associated with the consumption of vegetables, fruits, berries and meat [18], although in our sample the significant association between these subscales and meat consumption were only seen in the crude models. The more frequent intake of vegetables and fruit was also observed in 2–6-year-old British children who showed a higher pleasure in eating [36]. The food approaching appetitive traits are associated with the consumption of nutrient-rich foods and seem to be help children meet dietary recommendations. As seen in a study within the Generation XXI birth cohort, children with higher scores on Enjoyment of Food at 7 years showed better diet quality (measured through the Healthy Eating Index) 3 years later, indicating that children with this behavioural trait may have a well-balanced diet in nutritional terms, but may require food monitoring [26]. In other studies, food approaching appetitive traits have also been linked with more frequent meals (Food Responsiveness trait) [37] and a greater total energy and food intake in children (Enjoyment of Food and Food Responsiveness traits) [9]. High responsiveness to external stimuli, preference for foods high in fat and sugar and emotional eating are some behavioural aspects that could also explain the association between greater scores on food approach appetitive traits and child’s higher BMI [38, 39]. In this context, parental influences may be determinant for modelling children’s behaviour, not only through changes in food home environment, but also with their own eating behaviours and preferences [40]. In addition, parenting feeding styles can shape food consumption in childhood [41] and showed to have an impact on their BMI [42].

Regarding the relationship between emotional eating and food consumption, it seems that those children who change their food intake in response to negative emotions are more likely to consume unhealthy foods, such as salty snacks, energy-dense foods and sugar-sweetened beverages. It has been reported in previous studies with cross-sectional designs that Emotional Overeating was associated with lower consumption of high-fibre foods [18] and higher consumption of snacks, although the association was not observed after the 1-year follow-up [16]. Similar to our findings, in a sample of American adolescents, emotional eaters were more likely to consume both salty and sweet energy-dense foods, as well as sugar-sweetened beverages [43]. The greater consumption of these foods may contribute to an unbalanced energy intake of emotional eaters. As seen in previous studies, these appetitive traits have been positively associated with BMI and fat mass index in 10-year-old Dutch children [44], and waist-to-height ratio in a sample of Australian children with overweight/obesity [45].

In our study, the food approach behaviour Desire to Drink was positively associated with sugar-sweetened beverages and noncore foods consumption, and negatively associated with vegetables and fishery consumption 3 years later. The association with a more frequent intake of sugar-sweetened drinks was in line with a previous study by Sweetman et al. in the UK*,* which reported a higher consumption of sugar-sweetened beverages and low-calorie soft drinks in 11-year-old twins with higher scores on Desire to Drink [46]. Similar findings were described in a sample of Dutch children in a comparable age group as ours [16], and in another sample of preschoolers (only in the African–American subsample) [47]. Nevertheless, in one study with 6–8-year-old children this association was not established [18]. The higher consumption of energy-dense foods, also seen among children with higher scores on Desire to Drink, and the association between sugar-sweetened beverages consumption and the increased energy intake and body weight [48, 49], indicates the greater risk of future health implications. Previous studies also reported the relationship between this appetitive trait and a higher number of snacks consumed per day [18]. These findings suggest that these children show less interest in main meals which might explain the less frequent consumption of vegetables and fishery observed in our sample.

Our results showed that certain food avoidant traits, namely, Food Fussiness and Satiety Responsiveness, were prospectively associated with less frequent consumption of vegetables and fruit. In Generation XXI, a study has shown that 7-year-old fussy eaters (highly selective on foods) had lower scores on a Healthy Eating Index at 10 years of age [26]. These results seem to be in line with past research which was developed across different age groups [50, 51]. In particular, the association between picky eating in childhood (according to caregiver’s perception) and food consumption has been explored in a narrative review which summarised the results of 38 studies [50]. Similar to our findings, the majority of these studies observed a significantly lower consumption of vegetables in picky eaters compared with non-picky eaters; while regarding fruit consumption, 7 out of 13 studies reported lower consumption among picky eaters [50]. A systematic review and meta-analysis in children aged ≤ 30 months also reported that picky eating was associated with greater disliking of vegetables and fruit, although the evidence for consumption is more consistent for vegetables (5 of 5 studies), than for fruit (2 of 5 studies) [51]. Parental eating habits seem to play an important role, in particular the amount of fruit or vegetables consumed by parents which was a strong predictor children’s intake in previous study [36]. Another study with 9-year-old girls showed that mothers with a higher consumption of fruits and vegetables were less likely to pressure their daughters to eat and to have daughters who were picky eaters [52]. Regarding noncore foods, both reviews described mixed results, and only a limited number of studies found positive associations between fussy eating and consumption of noncore foods [50, 51]. As observed in our sample, the narrative review revealed that distinctive appetitive traits of picky eaters were linked with significantly lower consumption of fish. Authors also reported that most of the studies included in the review found differences in meat intake between picky eaters and non-picky eaters [50].

Interestingly, our study highlighted the association of two food avoidant traits, namely, Slowness in Eating and Satiety Responsiveness, with later greater milk consumption. These appetitive traits reflect children’s lack of interest in food and a reduction in eating rate as consequence [53]. A possible reason for the more frequent consumption of liquids, such as milk and sugar-sweetened beverages, among these children is their preference for drinks instead of solid foods, which are easier to consume [54], and usually accounts for most of their energy intake [55]. The association of food avoidant traits and higher milk consumption has been observed previously in younger children [56] and it seems to play an important role in the regulation of food consumption, mainly through a reduction in appetite at mealtimes [57]. In a study with 16-month-old children, higher scores on Satiety Responsiveness showed to be linked to the consumption of smaller meal sizes (defined as the amount of energy per eating occasions) [37]; while among 6–8-year-old children, Slowness in Eating was inversely associated with meat consumption [18], suggesting that these food items may have been substituted for snacks and milk, as observed in our sample.

### Strength and limits

Both data on child’s appetitive traits and food consumption was reported by the main caregiver, and this may be biased and potentially lead to an over report of healthy food intake due to social desirability. Nevertheless, the CEBQ showed good psychometric properties in our sample, besides being a validated tool, extensively used in other populations [58–60]. In addition, data collected with the FFQ was compared with 3-day food records in a sub-sample of children from Generation XXI at 4 and 7 years and showed acceptable validity [25]. Even so, the FFQ only gives information about the frequency of food consumption, and does not address consumed quantities. In our study, food consumption in childhood was categorized into groups based on the consumption frequency of certain foods in our sample. Adequacy of food intake was not analysed, due to the lack of official food group recommendations for this specific age. Despite these limitations, strengths of the present study include the prospective design, based on a birth cohort study with a large sample size, and the number of different food groups included in the analyses. Previous research on the association between appetitive traits and food consumption in school-aged children is scarce. This is the first study that prospectively examined these associations over a 3-year follow-up period for a wide range of food groups, and did not focus on specific food groups.

In conclusion, the results of the present study suggest that appetitive traits of 7-year-old children are associated with their food consumption at age 10. As we hypothesized, greater scores on food approach traits were prospectively associated with a more frequent consumption of energy-dense foods and salty snacks, besides being also linked to a greater consumption of vegetables and fruit 3 years later. In contrast, children with greater scores on food avoidant traits at the age of 7 consumed less frequently vegetables and fruit, and more frequently energy-dense foods and sugar-sweetened beverages 3 years later. In general, these appetitive traits were also associated with a more frequent consumption of milk when children were 10 years. Furthermore, our analysis suggests that part of the observed associations might be explained by the child’s food consumption at 7 years and/or by the appetitive traits at 10 years.

These findings highlight the need to assess children’s appetitive traits when providing dietary counselling, as an important tool to improve diet quality and normal growth. More longitudinal design studies are needed to help clarifying some of the associations, in particular those observed for milk, dairy products and sugar-sweetened beverages. Longer follow-up periods would also be helpful to better comprehend the long-term effects of appetitive traits on food consumption, including during adolescence and adult life.

### What is already known on this subject?

Evidence on how certain appetitive traits affect dietary consumption in school-aged children is supported by a limited number of prospective studies. The majority of prospective studies focused on a restricted group of foods, such as fruit and vegetables, which gather the stronger evidence, but other food groups, such as energy-dense foods, dairy products, starchy foods, meat and fishery, were less explored and findings are inconsistent.

### What this study adds?

Children’s appetitive traits at 7 years were associated with the consumption of several food groups at 10 years of age. Emotional Eating, Satiety Responsiveness and Food Fussiness were associated with worse dietary choices (in general, lower fruit and vegetables, and higher energy-dense foods and sugar-sweetened beverages consumption). These findings highlight the need to assess children’s appetitive traits when providing dietary counselling, as an important tool to improve diet quality and normal growth.

### Supplementary Information

Below is the link to the electronic supplementary material.

Supplementary file1 (DOCX 39 KB)

## Data Availability

The data sets generated during and/or analysed during the current study are available from the corresponding author on reasonable request.
