# Supplementary material for: Appetitive traits and food groups consumption in school-aged children: prospective associations from the Generation XXI birth cohort
Source: Eat Weight Disord. 2023 Aug 5;28(1):67. doi: 10.1007/s40519-023-01586-9 (PMC10404169; doi:10.1007/s40519-023-01586-9)
Supplement: Supplementary file 1 — Supplementary file1 (DOCX 39 KB) [file 40519_2023_1586_MOESM1_ESM.docx]

**Supplementary Table 1a.** Binary and multinomial logistic regressions for associations between child appetitive traits at 7 years and food consumption at 10 years of age (n=3806).

|  |  | **Eating behaviours at 7y** | | | | | | | |
| --- | --- | --- | --- | --- | --- | --- | --- | --- | --- |
| **Food consumption at 10y** |  | Enjoyment of Food | Food Responsiveness | Emotional Overeating | Desire to Drink | Satiety Responsiveness | Slowness in Eating | Emotional Undereating | Food Fussiness |
|  |  | OR (99% CI) | OR (99% CI) | OR (99% CI) | OR (99% CI) | OR (99% CI) | OR (99% CI) | OR (99% CI) | OR (99% CI) |
| **Vegetables ^a^** | 1.5-2.5 times/day | 1.14 (0.95-1.36) | 0.98 (0.82-1.17) | 1.00 (0.82-1.21) | 0.95 (0.82-1.10) | 0.97 (0.79-1.18) | 0.93 (0.79-1.09) | 1.02 (0.87-1.19) | 0.84 (0.69-1.03) |
| Ref: < 1.5 times/day | > 2.5 times/day | **1.27 (1.03-1.57)** | 1.07 (0.87-1.31) | 1.04 (0.83-1.30) | 0.89 (0.75-1.06) | 0.79 (0.63-1.00) | 0.86 (0.72-1.03) | 0.92 (0.77-1.10) | 0.86 (0.68-1.08) |
| **Fruit** | ≥ 2 times/day | **1.20 (1.03-1.41)** | 1.11 (0.95-1.30) | 1.06 (0.89-1.26) | 1.02 (0.90-1.16) | 0.93 (0.78-1.10) | 1.04 (0.91-1.20) | 0.98 (0.86-1.13) | 0.94 (0.80-1.12) |
| Ref: < 2 times/day |  |  |  |  |  |  |  |  |  |
| **Milk ^b^** | 1-2 times/day | 0.93 (0.77-1.14) | 0.97 (0.80-1.18) | 0.92 (0.74-1.15) | 1.04 (0.88-1.23) | 1.11 (0.88-1.39) | **1.27 (1.06-1.52)** | 0.98 (0.83-1.17) | 1.06 (0.85-1.32) |
| Ref: < 1 time/day | > 2 times/day | 0.89 (0.72-1.11) | 0.91 (0.74-1.13) | 0.91 (0.72-1.15) | 1.09 (0.91-1.30) | 1.21 (0.95-1.55) | **1.39 (1.14-1.68)** | 1.02 (0.84-1.22) | 1.06 (0.84-1.34) |
| **Yogurt** | 0.5-1 times/day | 0.98 (0.84-1.13) | 1.01 (0.88-1.18) | 1.05 (0.89-1.24) | 0.88 (0.78-1.00) | 0.91 (0.77-1.08) | 0.97 (0.85-1.10) | 0.98 (0.86-1.12) | 1.03 (0.88-1.21) |
| Ref: < 1 time/day |  |  |  |  |  |  |  |  |  |
| **Cheese** | 1-3 times/week | 1.00 (0.84-1.20) | 1.06 (0.89-1.26) | 1.06 (0.88-1.28) | 0.95 (0.82-1.10) | 1.06 (0.87-1.28) | 1.07 (0.92-1.25) | 1.01 (0.87-1.17) | 1.03 (0.83-1.27) |
| Ref: < 1 time/week | > 3 times/week | 0.94 (0.76-1.16) | 1.02 (0.83-1.26) | 1.12 (0.89-1.40) | 0.92 (0.77-1.09) | 1.13 (0.89-1.43) | 1.16 (0.96-1.40) | 1.10 (0.92-1.32) | 1.02 (0.82-1.27) |
| **Meat ^c^** | 2-4 times/week | 0.98 (0.83-1.16) | 1.01 (0.85-1.19) | 1.00 (0.83-1.20) | 1.03 (0.90-1.19) | 1.03 (0.85-1.24) | 1.02 (0.88-1.19) | 0.99 (0.86-1.15) | 1.08 (0.90-1.30) |
| Ref: < 2 times/week | > 4 times/week | 1.00 (0.83-1.20) | 1.05 (0.87-1.25) | 1.13 (0.93-1.39) | 1.07 (0.92-1.24) | 1.12 (0.91-1.37) | 1.15 (0.97-1.35) | 1.02 (0.87-1.20) | 0.99 (0.82-1.21) |
| **Fishery ^d^** | ≥ 3 times/week | 1.05 (0.91-1.22) | 0.96 (0.83-1.11) | 0.94 (0.80-1.10) | 0.93 (0.82-1.06) | 0.90 (0.77-1.07) | 0.96 (0.84-1.10) | **0.87 (0.77-0.99)** | 0.97 (0.82-1.14) |
| Ref: < 3 times/week |  |  |  |  |  |  |  |  |  |
| **Eggs** | > 1 time/day | 1.00 (0.86-1.17) | 0.97 (0.83-1.13) | 1.00 (0.84-1.18) | 0.98 (0.86-1.11) | 1.01 (0.85-1.20) | 0.97 (0.85-1.12) | 1.06 (0.93-1.21) | 1.05 (0.89-1.24) |
| Ref: ≤ 1 time/day |  |  |  |  |  |  |  |  |  |

Model adjusted for mother’s age, education, pre-pregnancy body mass index, child sex, sports practice, BMI z-score at 10y, food consumption at 7 years, and respective CEBQ subscale at 10 years.

^a^ Includes cooked or raw vegetables, and vegetable soup; ^b^ Includes milk and chocolate milk; ^c^ Includes all types of meats, excluding meat products; ^d^ Includes all types of fish and shellfish. Ref.: Reference category; OR: Odds Ratio; 99% CI: 99% Confidence Interval. Significant associations (p<0.01) are highlighted in bold-type.

.

**Supplementary Table 1b.** Binary and multinomial logistic regressions for associations between child appetitive traits at 7 years and food consumption at 10 years of age (n=3806) (cont.).

|  |  | **Eating behaviours at 7y** | | | | | | | |
| --- | --- | --- | --- | --- | --- | --- | --- | --- | --- |
| **Food consumption at 10y** |  | Enjoyment of Food | Food Responsiveness | Emotional Overeating | Desire to Drink | Satiety Responsiveness | Slowness in Eating | Emotional Undereating | Food Fussiness |
|  |  | OR (99% CI) | OR (99% CI) | OR (99% CI) | OR (99% CI) | OR (99% CI) | OR (99% CI) | OR (99% CI) | OR (99% CI) |
| **Meat Products ^e^** | 2-4 times/week | 0.98 (0.83-1.17) | 0.99 (0.83-1.18) | 0.99 (0.82-1.20) | 1.01 (0.88-1.17) | 1.05 (0.86-1.27) | 1.06 (0.91-1.23) | 1.00 (0.86-1.16) | 1.09 (0.90-1.31) |
| Ref: ≤ 2 times/week | > 4 times/week | 0.98 (0.81-1.20) | 1.01 (0.84-1.23) | 1.14 (0.92-1.41) | 1.03 (0.88-1.21) | 1.17 (0.95-1.46) | **1.21 (1.02-1.43)** | 1.06 (0.90-1.25) | 1.00 (0.82-1.24) |
| **Rice, potatoes and pasta** | ≥ 2 times/day | 0.86 (0.67-1.09) | 0.89 (0.70-1.13) | 0.89 (0.68-1.15) | 1.01 (0.82-1.24) | 1.07 (0.81-1.41) | 0.96 (0.77-1.19) | 1.03 (0.83-1.27) | 0.98 (0.75-1.28) |
| Ref: < 2 times/day |  |  |  |  |  |  |  |  |  |
| **Breakfast Cereals** | 2-5 times/week | 1.05 (0.87-1.26) | 1.17 (0.98-1.40) | 1.18 (0.96-1.45) | 1.06 (0.90-1.23) | 1.05 (0.85-1.29) | 1.03 (0.87-1.21) | 0.99 (0.84-1.16) | 0.95 (0.77-1.15) |
| Ref: < 2 times/week | ≥ 6 times/week | 1.11 (0.92-1.34) | 1.19 (0.99-1.43) | 1.14 (0.93-1.40) | 1.07 (0.92-1.24) | 0.99 (0.80-1.21) | 0.96 (0.82-1.13) | 1.02 (0.87-1.20) | 0.93 (0.77-1.14) |
| **Sugar-sweetened beverages ^f^** | 0.5-1 times/day | 0.90 (0.75-1.09) | 0.83 (0.69-1.00) | 0.81 (0.66-1.00) | 0.96 (0.82-1.13) | 1.16 (0.94-1.43) | 1.03 (0.88-1.22) | 1.12 (0.95-1.31) | 0.95 (0.77-1.16) |
| Ref: < 0.5 times/day | > 1 time/day | 0.89 (0.74-1.07) | 0.83 (0.69-1.00) | 0.95 (0.77-1.16) | 1.00 (0.85-1.17) | 1.01 (0.82-1.25) | 1.03 (0.87-1.21) | 1.02 (0.87-1.20) | 0.98 (0.80-1.20) |
| **Energy-dense foods ^g^** | 1.3-2 times/day | 0.89 (0.75-1.06) | 0.94 (0.79-1.12) | 1.09 (0.90-1.31) | 1.02 (0.88-1.18) | 0.99 (0.82-1.20) | 1.03 (0.88-1.20) | 1.11 (0.95-1.29) | 1.05 (0.87-1.26) |
| Ref: < 1.3 times/day | > 2 times/day | 0.93 (0.78-1.12) | 0.98 (0.82-1.18) | 1.06 (0.87-1.30) | 1.12 (0.96-1.30) | 1.13 (0.92-1.39) | 1.04 (0.88-1.22) | 1.06 (0.90-1.24) | 1.06 (0.87-1.30) |
| **Salty snacks ^h^** | 0.6-1 times/day | 0.98 (0.82-1.17) | 1.05 (0.89-1.25) | 1.07 (0.88-1.29) | 1.00 (0.87-1.16) | 0.88 (0.73-1.07) | 0.89 (0.77-1.04) | 1.02 (0.88-1.19) | 1.06 (0.88-1.27) |
| Ref: < 0.6 times/week | > 1 time/day | 0.96 (0.80-1.15) | 1.01 (0.85-1.21) | 1.12 (0.92-1.36) | 1.09 (0.94-1.26) | 0.89 (0.73-1.08) | 0.94 (0.80-1.10) | 1.07 (0.91-1.24) | 1.15 (0.95-1.39) |

Model adjusted for mother’s age, education, pre-pregnancy body mass index, child sex, sports practice, BMI z-score at 10y, food consumption at 7 years, and respective CEBQ subscale at 10 years

^e^ Includes ham, sausages and smoked meat products; ^f^ Includes carbonated and non-carbonated sugar-sweetened beverages; ^g^ Includes pizza, burgers, chips, chocolate, snacks, cakes, pastry, cookies and candies; ^h^ Includes meat and fish based fried snacks. Ref.: Reference category; OR: Odds Ratio; 99% CI: 99% Confidence Interval. Significant associations (p<0.01) are highlighted in bold-type.
